# An On-Station Analysis of Factors Affecting Growth Traits of Pure Red Maasai and Dorper Sheep Breeds under an Extensive Production System

**DOI:** 10.3390/ani13020300

**Published:** 2023-01-15

**Authors:** George Wanjala, Nelly Kichamu, Ludovic Toma Cziszter, Putri Kusuma Astuti, Szilvia Kusza

**Affiliations:** 1 Centre for Agricultural Genomics and Biotechnology, H-4032 Egyetem tér 1, 4032 Debrecen, Hungary; 2 Doctoral School of Animal Science, University of Debrecen, H-4032 Böszörményi út 138, 4032 Debrecen, Hungary; 3 Ministry of Agriculture, Livestock, Fisheries, Cooperatives and Irrigation, Directorate of Livestock Production, Bungoma P.O. Box 437-50200, Kenya; 4 Ministry of Agriculture Livestock, Fisheries and Cooperatives, State Department of Livestock Development, Naivasha Sheep and Goats Breeding Station, Naivasha P.O. Box 2238-20117, Kenya; 5 Bioengineering Faculty of Animal Resources, Banat’s University of Agricultural Sciences and Veterinary Medicine “King Michael I of Romania”, Calea Aradului 119, 300645 Timisoara, Romania

**Keywords:** weaning weight, yearling weight, growth traits, non-genetic factors, extensive production

## Abstract

**Simple Summary:**

One of the indigenous sheep breeds that is primarily produced in Kenya under an extensive production system is the Red Maasai sheep. It has a reputation for being able to thrive in arid and semi-arid environments. A common belief is that, when contrasted with the Dorper breed, it performs poorly in terms of growth traits. Therefore, to enhance their growth traits, the Red Maasai are randomly crossed with Dorper. In contrast, the Red Maasai sheep breed outperformed the Dorper sheep breed in this study in terms of post-weaning growth rate, once more demonstrating early resilience and adaptation to local environmental conditions independent of maternal influence.

**Abstract:**

This comparative study aimed to evaluate genetic and environmental factors’ effects on the growth traits of lambs in Dorper and Red Maasai (RedM) sheep breeds. The data analyzed contained the following measurements: birth weight (Bwt), weaning weight (Wwt), yearling weight (Ywt), birth type (single or twins), and details on each lamb’s dam (dam ID and age) and sire. Except for the RedM, whose birth weight decreased with time, both breeds generally showed an increase in other growth weights across the study period, with yearly variations affecting both breeds. Additionally, analysis by a linear mixed model with restricted maximum likelihood (REML) showed that only breed as a factor significantly (*p* < 0.05) influenced birth weight; breed, sex, and birth type all significantly (*p* < 0.05) influenced weaning weight, whereas season, sex, and dam age significantly (*p* < 0.05) influenced yearling weight. The RedM breed outperformed the Dorper breed in post-weaning growth rate, demonstrating early resilience and adaptation to local environmental factors independent of maternal influence. Breed and non-genetic factors play a vital role in the growth of lambs, and the results of this study offer an opportunity for improved farm management under an extensive production system and selection for the conservation of the indigenous Red Maasai breed.

## 1. Introduction

Small ruminants in Kenya contribute significantly to the Kenyan national economy, with a value of USD 688 million USD in 2020, up from USD 469 million in 2016 [1]. Furthermore, Kenya has recorded an increase in the sheep population from 16.7 million heads in 2015 to 27.4 million in 2019 [2], indicating their economic importance to the Kenyan economy.

More than 60% of the sheep populations in Kenya are native and predominantly kept under an extensive farming system, majorly by pastoralists and resource-poor farmers [3,4]. Their production environments are mainly characterized by harsh environmental conditions such as arid and semi-arid regions, poor-quality pasture and high exposure to parasites. Indigenous sheep breeds are tolerant of such harsh conditions [5], giving them an advantage over exotic breeds.

In Kenya, sheep breeds are mainly classified as either fat-tailed, which includes the Red Maasai (RedM) and East African fat-tailed breeds [6], or fat-rumped, which includes blackhead Somali sheep. The RedM is predominant among other sheep breeds; it is spread across the country, followed by the East African fat-tailed breed. RedM is the most preferred by the pastoralist communities due to its resistance to endoparasites, particularly *Haemonchus contortus* [4], its tolerance to drought [7], and its ability to walk long distances in search of pasture and water. This breed’s reproductive and growth performance is rated lower than those of the exotic Dorper breed.

The Dorper sheep breed, which originated from South Africa, is a hair meat sheep [8,9] and a hardy composite breed known for superior growth performance, with quality carcass traits at a relatively early stage of growth [4]. The development of Dorper sheep in South Africa was necessitated by the need for a sheep breed with high reproductive efficiency in the arid and extensive production systems in their native country [10]. Similarly, the desire to improve the productivity of the pure RedM breed led to the importation of the Dorper breed in the mid-1970s. Farmers crossbred their sheep with Dorper to improve growth performance [5]. However, continuous indiscriminate crossbreeding has endangered the existence of the novel indigenous RedM genes.

As a result, the Kenyan government established sheep and goat farms in some parts of the country in different ecological zones to preserve the most vulnerable indigenous small ruminant breeds, particularly the RedM sheep breed. The main mandate of these farms is to objectively select and breed pure RedM to sell breeding stock/rams to farmers to upgrade their stocks. Alongside RedM, Dorper sheep are also bred, and farmers use Dorper rams to crossbreed with local RedM for the improved growth performance and meat traits of the progeny. In this case, Dorper rams are used as terminal sires, with the assumption that all the lambs are slaughtered to control native gene erosion.

To our knowledge, this is the first study aiming to establish the phenotypic trends and the correlation between quantitative phenotypes as well as examine the breed and non-genetic factors affecting the growth performance of the Dorper and the indigenous RedM breeds kept at the Naivasha sheep and goats station farm in Kenya. The results of this study will provide an opportunity for improved flock management under an extensive management system for an improved in situ indigenous sheep conservation plan in Kenya.

## 2. Materials and Methods

### 2.1. Source of Data, Flock Management and Environmental Information

**Figure 1:**
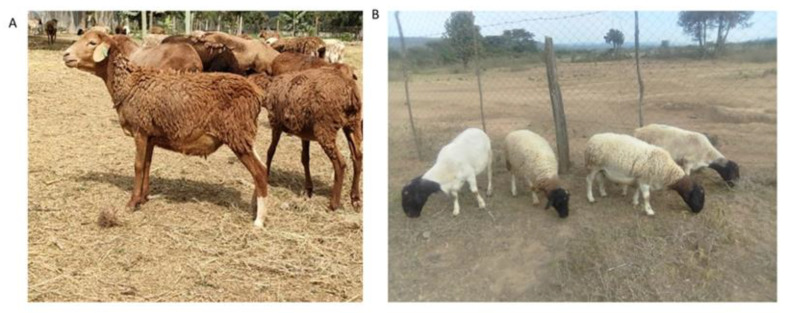
RedM (**A**) and Dorper (**B**) sheep.

Data used in the present study were obtained from the Naivasha sheep and goat station (Ol’magogo farm) located at latitude −0.551494 and longitude 36.413791. The station is managed by the government of Kenya under the Ministry of Agriculture, Livestock, Fisheries and Cooperatives within the State Department of Livestock Development. To improve the productive potential of the indigenous sheep breeds, particularly RedM, the government of Kenya, in collaboration with the Food and Agricultural Organization (FAO), initiated a genetic improvement program in the 1970s with a focus on within-breed selection and crossbreeding between the RedM and Dorper (Figure 1) breeds to combine desirable traits in both breeds [11].

Ol’Magogo is in Kenyan ecological zone IV, which is classified as semi-arid. Historically, the region experiences a bimodal rainfall pattern, with March–May being the long-wet period, while October–December is the short-wet period. On average, the region receives a poorly distributed rainfall of 680 mm per annum. The average minimum and maximum temperatures range between 8 °C and 28 °C, respectively, with a relative humidity of between 60% and 70%. The elevation of this region ranges between 1830 and 2330 m a.s.l. (meters above sea level). The natural vegetation is predominantly star grass (*Cynodon plectostachyus*) with scattered tall acacia trees (*Acacia xanthophyloea*). The soil is volcanic in origin, alkaline (pH 7.4), sodic and deep.

The animals were raised under an extensive production system where they were grazed in paddocks on natural pastures during the day, with average grazing hours of 8 per day, and enclosed at night. At a stocking density of nine sheep per hectare, flocks were grazed separately; hence, the chances of mixing or crossbreeding were minimized, and none of the breeds received preferential treatment. Therefore, performance differences between the breeds are assumed to be genetically driven. Selection for breeding was based on pure-breed typical conformation and animal live weight. Ewes were joined to rams depending on their pre-tupping weight (Pwt). Generally, ewes weighing below 25 kg were not joined. The farm has two mating seasons: October–November and April–May, which leads to lambing in August–September and February–March, respectively. The age at first joining was 12 to 18 months, and longevity depended on the reproductive performance of the animal. Lambs were left to run freely with their mothers until weaning (90 to 120 days of age). Feed supplementation was seldom done, even in nursing dams. However, mineral-licking blocks were provided. Water was also provided ad libitum. Disease and parasite management measures were mainly prophylactic and biosecurity oriented. Animals were routinely weighed at birth, weaning, yearling and before breeding. Flushing by grazing ewes on regenerative pasture was done one month before breeding season to improve the breeding flock’s body condition score, as indicated in [12,13,14].

### 2.2. Description of Data

The Ol’magogo Sheep and Goat Station in Naivasha, Kenya provided the 903 growth performance records for the Dorper and RedM sheep breeds, which were evaluated for the years 2007 through 2020. Data collection was done as part of station record-keeping procedures, not for the goal of this study. The information on growth performance includes the lambs’ birth weight (Bwt) at birth, weaning weight (Wwt) on the 90th day after birth, and yearling weight (Ywt) on the 365th day after birth. The average daily gains (ADG1) and (ADG2) before and after weaning, respectively, were subsequently determined. Additionally, each lamb’s sire and dam were matched. The dam’s age and her tupping weight (Pwt) were also included. Table 1 presents an overview of the data evaluated. More information on the data used in this study is presented in Table S1.

### 2.3. Statistical Analysis

Phenotypic trends of lambs’ weights (Bwt, Wwt and Ywt) over the years between 2007 and 2020 were plotted using the ggplot2 package. Further average daily gain pre-weaning (90 days post lambing) and post-weaning (275 days after weaning) were compared between both breeds using the Wilcoxon test [15] implemented in the ggpubr [16] package.

The linear model [17] procedure in R studio from [18] was used to establish the effects of potential fixed factors on growth traits using the lme4 package [19]. The effects of random factors were assessed before fitting linear mixed models. Random factors, which included year, sire, dam, tupping weight, and individual lamb differences, were fitted against each trait. In the subsequent model testing, only random variables with a significant effect were considered. Model testing under the maximum likelihood estimation (ML) [20] method was performed on eight models, which ignored all random factors using a simple linear regression model, while linear mixed models were fitted with different combinations of random factors. The model that yielded the lowest Akaike information criterion (AIC) [21] value per trait was considered for that particular trait. The selected model was then refitted using the restricted maximum likelihood (REML) [22] method. Tests and significant level effects of each factor on each trait were performed using the lmerTest package [23]. Only fixed factors with a significant effect on the trait were selected for the further refitting of the model and the evaluation of the factor differences under REML. The fixed effects included in the model for different traits at the model testing phase were breed, the season of birth, the sex of the lamb, the dam’s age at lambing, and birth type (singleton or twin). The model did not include collinear variables to avoid the false non-significance of potential main effect factors. Table 2 presents a summary of the models tested for each trait. Model 2 was considered for all traits since it produced the lowest AIC of all the traits.

## 3. Results

### 3.1. Phenotypic Trends

**Figure 2:**
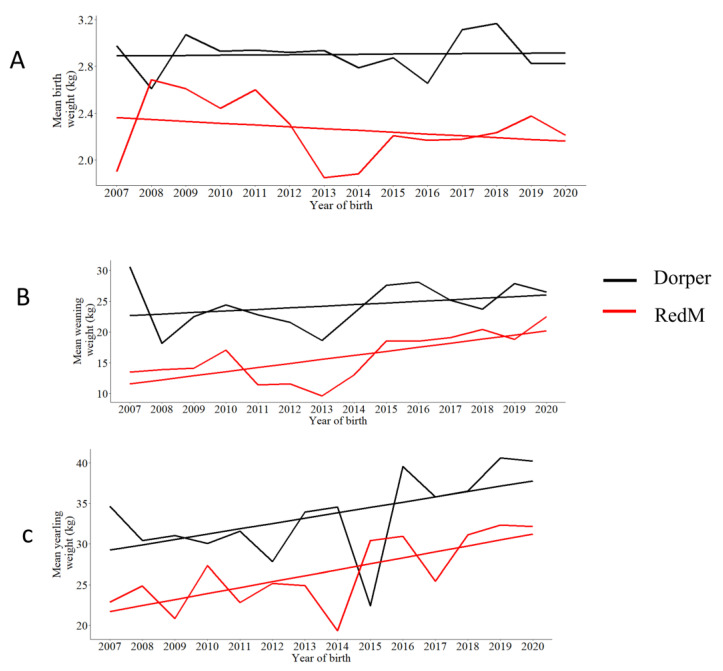
Comparative phenotypic trends for growth weights. (**A**) Phenotypic trend over the years for birth weight; (**B**) phenotypic trend over the years for weaning weight; (**C**) phenotypic trend over the years for yearling weight.

**Figure 3:**
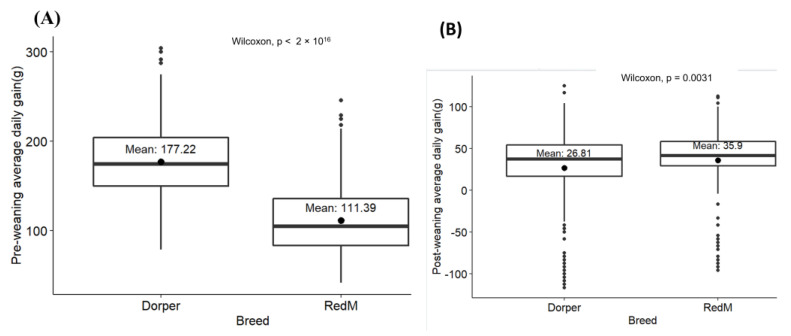
Comparison of average growth rate. (**A**) Pre-weaning growth rate; (**B**) post-weaning growth rate. The point inside the box plot shows the overall mean for the specific breed. Additionally, the *p* values are shown on the upper side of the figure.

The phenotypic trends for each growth trait under investigation were analyzed and are presented in Figure 2A–C. Generally, the Dorper breed registered an increase in birth weight over the years, while the RedM breed’s mean birth weight declined over the same period. However, both breeds registered an increase in weaning and yearling weights over the same period of study. Nevertheless, despite the general trends highlighted above, there is an observable fluctuation of growth weights across the years. Expectedly, the Doper sheep seem to be better than the RedM sheep in both Bwt, Wwt and Ywt. Furthermore, Dorper lambs appeared to gain weight faster than the RedM lambs before weaning, whereas the RedM lambs grow slightly faster than the Dorper lambs post-weaning, as shown in Figure 3A,B.

### 3.2. Factors Affecting Growth Performance

**Figure 4:**
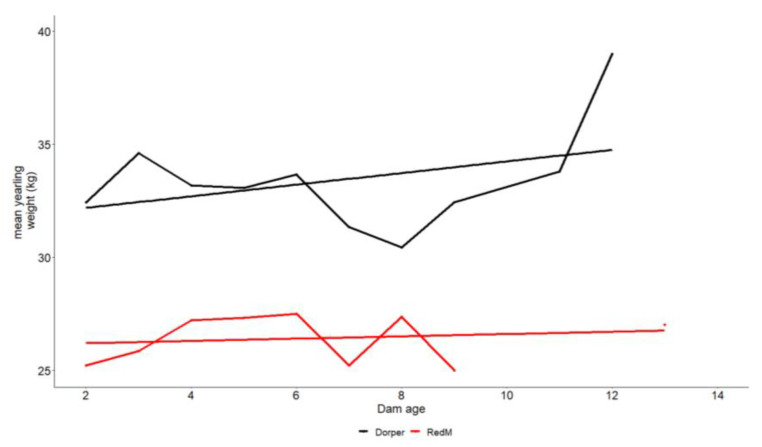
Effect of dam age on the yearling weight.

A summary of the fixed effects, their significance, and their corresponding residual standard deviations are shown in Table 3. Breed was the only factor that significantly influenced birth weight, whereas sex, birth type, and breed all significantly (*p* < 0.05) influenced weaning weight. On the other hand, the yearling weight was significantly (*p* < 0.05) influenced by the season, sex, and the dam’s age. Dam age was regarded as a continuous variable in this instance. The mean yearling weight increased with an increase in dam age, as presented in Figure 4. Males were statistically (*p* < 0.05) heavier than females, and singletons were significantly (*p* < 0.05) heavier than twins at weaning. Dorper lamps were significantly (*p* < 0.05) heavier than RedM lambs at birth (Table 4), and they were also heavier than RedM lambs at weaning. Compared to lambs born in the September–October lambing season, lambs born in the March–April lambing season were significantly (*p* < 0.05) heavier.

## 4. Discussion

### 4.1. Phenotypic Trends

In general, both breeds showed an increase in all growth weights, with yearly variances affecting both breeds (Figure 2, Figure 3 and Figure 4), except for the RedM regarding birth weight, which declined over time. Because of the effects of climate change, it should be considered that the weather pattern was not constant over the study period. Therefore, fluctuations in Kenyan annual weather patterns [25] may also have contributed to variations in sheep growth weights. The RedM appears to increase in weaning weight faster than the Dorper breed, but both breeds increase in yearling weight at roughly the same rate. The phenotypic trend observed in the present study is comparable to the one observed in the study conducted by [4]. Dorper sheep grew significantly faster before weaning than RedM sheep did. This growth rate is possibly attributed to their good genetic potential for growth rate and mothering ability [26]. Unexpectedly, the RedM breed performed better than the Dorper breed in the post-weaning growth rate, indicating a better survival of lambs without maternal care, an indicator of early adaptation to the environment [27]. The RedM breed is said to be more resistant to nematodes, particularly *Haemonchus contortus* [28,29,30,31], and more tolerant to heat stress [6] than Dorper sheep. It is worth mentioning here that the performance of Dorper sheep was superior to the Red Maasai in almost all growth traits except the post-weaning growth rate. We assume that prophylactic and periodic biosecurity measures for disease and pest/parasite management being offered at the station could have sustained the performance of Dorper sheep over the years. However, it can also be argued that, since Dorper sheep were imported in the 1970s, possibly, the present generation could perform better than the indigenous sheep under a similar management level. This assumption can be confirmed or invalidated by the genomic analysis of signatures of selection for adaptation alongside the RedM sheep. König [7] recommended that Dorper sheep offer a good opportunity to improve the performance of the local breeds by crossbreeding, as the hybrid offspring will quickly adapt to the environment, as well as be superior in production to their parents due to hybrid vigor.

### 4.2. Factors Affecting Growth Performance

Surprisingly, only breed had a significant effect on birth weight among all the supposedly fixed-effect components. Dorper lambs were significantly heavier than the RedM lambs by 0.58 kg. This could be due to the larger mature size of the Dorper breed [5] compared to the RedM. Weaning weight was significantly affected by breed, sex, and birth type. Dorper lambs once again were significantly heavier than the RedM sheep, agreeing with several studies, e.g., [4,7,32]. The Dorper breed is a fast-growing breed with a large body size compared to the RedM breed. Large body size is an important indicator of a good growth rate [5]. However, it might not be a good indicator of the dressing percentage of the carcass. Studies conducted on the performance of indigenous sheep breeds X Dorper lambs indicated superior growth performance of the crossbred lambs compared to the pure-breed parents in all traits [4,26,33] because of heterosis. Therefore, it would be advisable to enhance crossbreeding with Dorper terminal sires. As a result, all the lambs produced would be heavier than their parents and should all be slaughtered to evade the risks of genetic erosion of pure-bred Red Maasai sheep. It is important to note here that breed as a factor did not have a significant influence on the yearling weight, indicating that the RedM weight on the 365th day post-lambing was not significantly different from Dorper lambs.

The sex of lambs also significantly influenced the variation of Wwt and Ywt. Males were generally heavier than females at weaning and the first 365 days of life. This indicates the existence of sexual dimorphism in both breeds, and these results are comparable to the previous findings such as [34,35,36]. Similarly, birth type as a factor also had a significant effect on the Wwt. Singletons were significantly heavier than twins. The findings in this study are comparable to the results reported in [4,37,38], where in both studies, singletons were heavier than lambs resulting from multiple births. Therefore, in this case, it would be advisable to enhance the management of dams with multiple lambs for improved flock productivity.

Another significant factor that influenced the Ywt was the lambing season. On the 365th day of life, lambs born in April/May were considerably (*p* < 0.05) heavier than lambs born in the September/October lambing season. However, the season has no influence on lamb Bwt and Wwt. The long wet season, which lasts until August in the study area, coincides with the lambing season in April/May. The sheep flock would be able to meet their nutritional needs, as there is an abundance of fresh foliage during this time. Lambs would consequently grow more quickly. The long wet season is immediately followed by the short wet season (October-December), which is then followed by a dry period (December-February), and hence, lambs born in September/October are weaned during the dry period, which is characterized by heat stress and a lack of adequate feed quality and quantity, which results in a low growth rate of lambs [39]. Therefore, it is critical to design a comprehensive farm breeding strategy to optimize the use of climatic variability and/or the use of technology to sustainably conserve feed resources for use during the scarcity of naturally available fresh fodder.

## 5. Conclusions

The RedM lambs’ higher post-weaning performance may be an indication of early resilience and adaptation to local environmental variables independent of maternal influence. This underlines once more the fact that native breeds of sheep cannot be entirely replaced by foreign breeds, notwithstanding the desire for superior growth performance in exotic breeds. This study’s findings indicate that there may be a genetic benefit from within-breed selection, which is consistent with Kenya’s efforts to maintain the native RedM breed. Dorper sheep should only be used as terminal sires to take advantage of heterosis. The breeding program should be planned to maximize the growing performance of the lambs while considering any effects of potential fixed factors on the desired trait. The establishment of a better and more precise conservation effort should also include genetic diversity and genomic research on the probable genomic variations underlying phenotypic performance, including adaptation. The nutritional profile of the available natural pasture should be defined at the paddock level to ensure proper pasture usage.

## Figures and Tables

**Table 1:** Summary of the data analyzed in this study.

Breed	No. of Lambs	No. of Ewes	No. of Sires
Dorper	422	187	7
RedM	481	208	6
Total	903	395	13

**Table 2:** A summary of models tested with their respective AIC values per trait.

Models Fitted		AIC Values		
Model	Equation	Bwt	Wwt	Ywt
1	Y = X_a_ + Z1_b_ + Z2_c_ + Z3_d_ + Z4_f_ + e	1194.360	5396.427	5929.867
2	Y = X_a_ + Z1_b_ + Z2_c_ + Z3_d_ + Z4_f_ + e + u_s,y,p_	1112.053	4915.532	5422.159
3	Y = X_a_ + Z1_b_ + Z2_c_ + Z3_d_ + Z4_f_ + e + u_s,y_	1135.761	4929.478	5495.143
4	Y = X_a_ + Z1_b_ + Z2_c_ + Z3_d_ + Z4_f_ + e + u_s,p_	1119.153	4940.065	5620.322
5	Y = X_a_ + Z1_b_ + Z2_c_ + Z3_d_ + Z4_f_ + e + u_y,p_	1174.957	5056.327	5739.314
6	Y = X_a_ + Z1_b_ + Z2_c_ + Z3_d_ + Z4_f_ + e + u_s_	1137.907	4962.471	5682.721
7	Y = X_a_ + Z1_b_ + Z2_c_ + Z3_d_ + Z4_f_ + e + u_y_	1181.524	5078.336	5797.795
8	Y = X_a_ + Z1_b_ + Z2_c_ + Z3_d_ + Z4_f_ + e + u_p_	1191.214	5350.029	5843.383

Where Y is a vector of observations on the specific trait of the lamb, while “a, b, c, d” and “f” are vectors of fixed effects influencing the trait. They included breed, season, sex, birth type and dam’s age at lambing. Here, “e” is a vector of residual errors while “u” represents the random factors sire (s), year (y), and tupping weight (p). The least-square means were obtained using lsmeans package [24] in R studio.

**Table 3:** Effect significance of each factor on a specific growth trait.

Growth Traits/Factors	Breed	Season	Sex	Birth Type	DAM AGE	Residual Std Dev
Birth weight	***	-	-	-	-	0.43
Weaning weight	***	-	***	**	-	3.45
Yearling weight	-	***	***	-	**	4.33

Significance: ***, *p* = 0.000; **, *p* = 0.01.

**Table 4:** Factor difference per trait.

Fixed Effects	Bwt	Wwt	Ywt
	LSM ± SE	LSM ± SE	LSM ± SE
Breed (*p*-value)	(0.0001)	(<0.0001)	
Dorper	2.90 ± 0.072	24.2 ± 1.10	-
RedM	2.32 ± 0.0697	15.9 ± 1.09	-
Season of birth (*p*-value)			(0.0003)
April–May	-	-	31.1 ± 2.99
Sept–Oct	-	-	29.8 ± 2.99
Sex (*p*-value)		(<0.0001)	<0.0001
Female	-	19.6 ± 0.987	29.8 ± 2.99
Male	-	20.6 ± 0.985	31.0 ± 2.99
Birth type (*p*-value)		(0.0099)	
Single	-	20.5	-
Twin	-	19.6	-

The empty cells (-) indicate that the factor was excluded from the final model since it did not have a significant effect on the trait. The residual standard errors for the models used to generate least square means (LSM) and their associated standard errors are presented in Table 3.

## Data Availability

The raw data analyzed in this study are available upon request from the corresponding author.

## Supplementary Materials

The following supporting information can be downloaded at: https://www.mdpi.com/article/10.3390/ani13020300/s1: Table S1: A summary of traits per year per breed for the data used in this study.
